# Remarkably high mobility ultra-thin-film metal-oxide transistor with strongly overlapped orbitals

**DOI:** 10.1038/srep19023

**Published:** 2016-01-08

**Authors:** Chen Wei Shih, Albert Chin, Chun Fu Lu, Wei Fang Su

**Affiliations:** 1 Department of Electronics Engineering, National Chiao Tung University, Hsinchu 300, Taiwan; 2 Depatment of Materials Science & Engineering National Taiwan University, Taipei 10617, Taiwan

## Abstract

High mobility channel thin-film-transistor (TFT) is crucial for both display and future generation integrated circuit. We report a new metal-oxide TFT that has an ultra-thin 4.5 nm SnO_2_ thickness for both active channel and source-drain regions, very high 147 cm^2^/Vs field-effect mobility, high I_ON_/I_OFF_ of 2.3 × 10^7^, small 110 mV/dec sub-threshold slope, and a low V_D_ of 2.5 V for low power operation. This mobility is already better than chemical-vapor-deposition grown multi-layers MoS_2_ TFT. From first principle quantum-mechanical calculation, the high mobility TFT is due to strongly overlapped orbitals.

The metal-oxide thin-film transistor (TFT)^1^,^2^,^3^,^4^,^5^,^6^,^7^,^8^,^9^,^10^,^11^,^12^,^13^,^14^,^15^,^16^,^17^,^18^,^19^,^20^ is a revolutionary technology for displays due to its high mobility and simple process. The high mobility of zinc-oxide (ZnO)-based materials was attributed to the spatially spread metal ns orbitals with isotropic shape, which is possible to overlap the neighboring metal ns orbitals^7^. However, the high performance ZnO-based TFTs of InGaZnO^7^, bi-layer InSnO/InGaZnO^11^, InZnO^13^, and GaZnON^18^ compounds usually contain Indium (In) or Gallium (Ga), which are rare elements in earth’s crust. In addition, device performance is sensitive to the moisture degradation and atomic composition of these compound. Alternatively, high mobility, high transistor on-current (*I*_*ON*_), and low off-current (*I*_*OFF*_) TFTs are found in two-dimensional metal-chalcogenide^21^,^22^,^23^,^24^,^25^,^26^,^27^,^28^. However, chemical vapor deposition (CVD)-grown MoS_2_^25^,^26^,^27^,^28^ shows a considerably lower mobility compared with peeled-off flakes from crystals^21^. To further increase the display pixel density and drive organic light-emitting diodes (OLED), higher mobility and *I*_*ON*_ than those of ZnO-based TFTs are needed. The low DC and switching power consumptions are other technological trends for displays that require a low *I*_*OFF*_ and low operation voltage. In this study, a remarkably high field–effect mobility (*μ*_*FE*_) of 147 cm^2^/Vs was demonstrated experimentally in tin-oxide (SnO_2_) TFT. This TFT also showed a high *I*_*ON*_*/I*_*OFF*_ of 2.3 × 10^7^, low sub-threshold swing (*SS*) of 0.11 V/decade, low threshold voltage (*V*_*T*_) of 0.27 V, low drive voltage of 2.5 V for low switching power, and ultra-thin layer with a thickness of 4.5 nm. Such ultra-thin thickness is comparable with that of multilayered MoS_2_^27^ for low DC standby power consumption. Notably, Sn (Group IV) has ns^2^np^2^ electron configuration and directive sp^3^ orbitals, which differ from those of Zn^7^. According to first principle quantum-mechanical calculations, the considerably high *μ*_*FE*_ in SnO_2_ TFT is caused by its overlapped s-orbitals even in an ns^2^np^2^ configuration.

## Results

**Figure 1:**
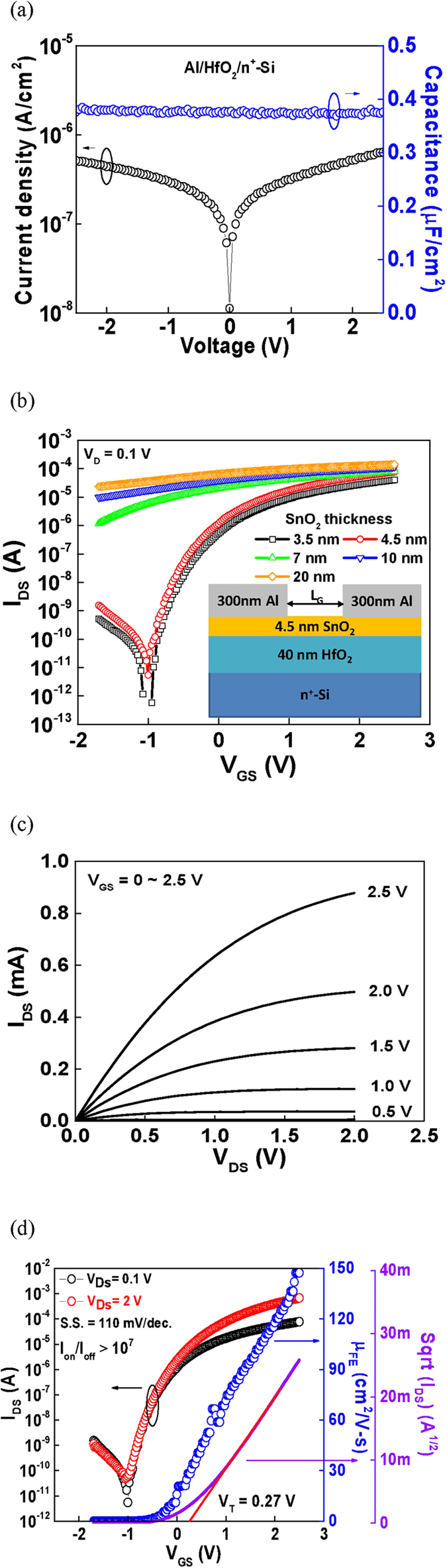
(**a**) *I-V* and *C-V* characteristics of gate capacitor, (**b**) *I*_*DS*_*-V*_*GS*_characteristics of Al/SnO_2_/HfO_2_/n^+^-Si TFTs with 20 ~ 3.5 nm SnO_2_ layers, (**c**) *I*_*DS*_*-V*_*DS*_ and (**d**) *I*_*DS*_*-V*_*GS*_, *μ*_*FE*_*-V*_*GS*_ and Sqrt(*I*_*DS*_)*-V*_*GS*_ characteristics of Al/SnO_2_/HfO_2_/n^+^-Si TFTs at 4.5 nm SnO_2_ thickness. The gate length is 50 μm.

To increase the transistor *I*_*ON*_ and reduce the operation voltage, a high-dielectric-constant (high-κ) gate insulator^12^,^15^ was used for the TFT. Figure 1(a) shows the current-voltage (*I-V*) and capacitance-voltage (*C-V*) characteristics of a gate capacitor with top Aluminum (Al) electrode, high-κ hafnium-oxide (HfO_2_), and bottom n^+^-Si. In the Al/HfO_2_/n^+^-Si capacitor, a small leakage current of 5.7 × 10^−7^ A/cm^2^ at 2 V was obtained at a capacitance density of 0.38 μF/cm^2^. The high capacitance density yielded a low equivalent-oxide-thickness (EOT) of only 9.1 nm, which was due to the high-κ HfO_2_ with a κ of 17. Figure 1(b) shows the transistor’s drain current versus gate voltage (*I*_*DS*_*-V*_*GS*_) characteristics of the SnO_2_/HfO_2_ TFTs with 4.5 ~ 20 nm thick SnO_2_. The device with thick 20 nm SnO_2_ failed to show proper pinch off *I*_*OFF*_ due to very high conductivity, although the device has very high *I*_*ON*_. The device with 4.5 nm thick SnO_2_ shows the best *I*_*ON*_*/I*_*OFF*_ performance. Figure 1(c,d) show the transistor’s drain current versus drain voltage (*I*_*DS*_*-V*_*DS*_), *I*_*DS*_*-V*_*GS*_, and *μ*_*FE*_*-V*_*GS*_ characteristics of the SnO_2_/HfO_2_ TFT with 4.5 nm thick SnO_2_, respectively. The device was operated in the enhancement mode of an n-channel metal-oxide-semiconductor field-effect transistor (nMOSFET) at a low operation voltage of 2.5 V. The device also showed a high *I*_*ON*_*/I*_*OFF*_ of 2.3 × 10^7^, low *SS* of 110 mV/decade, and low *V*_*T*_ of 0.27 V. The *V*_*T*_ was extracted from the intercept of the linear *I*_*DS*_^1/2^*-V*_*GS*_ curve in a saturation region. A high *I*_*ON*_ is crucial to drive the OLED and increase the display pixel density, whereas a low *I*_*OFF*_ is required to reduce the DC standby power. The low *SS* with the mean value and standard deviation of 100.2±19.4 mV/decade indicates the good oxide/semiconductor interface to turn on the transistor fast. The *I*_*ON*_ showed an inversely proportional relation with gate length in a wide gate length TFT, a typical method to extract mobility correctly for Si MOSFET and metal-gate/high-κ MOSFET^29^,^30^,^31^. A remarkably high *μ*_*FE*_ of 147 cm^2^/Vs is obtained with the mean value and standard deviation of 141.6 ± 11.5 cm^2^/Vs, which is higher than that of ZnO-based TFTs^3^,^4^,^5^,^6^,^7^,^8^,^9^,^10^,^11^,^12^,^13^,^14^,^15^,^16^,^17^,^18^,^19^,^20^ and even higher than that of a CVD-grown multilayered MoS_2_ MOSFET^21^,^22^,^23^,^24^. To reach high mobility, epitaxial growth of crystalline MoS_2_ on a crystal substrate is needed. Unfortunately, the mobility is lower for CVD-grown MoS_2_^25^,^26^,^27^,^28^ than peeled-off flakes from crystals^21^,^22^,^23^,^24^,^25^,^26^,^27^,^28^. The lattice mismatch caused defects are the other major concern for circuit yield. In contrast, high mobility SnO_2_ TFT is achievable on the amorphous substrate and free from lattice-mismatch defects. Such metal-oxide has already been used to manufacture TFT circuit for display. It is crucial to notice that the mobility of metal-oxide increases with increasing carrier density^7^. In the 4.5-nm-thick SnO_2_ TFT, the high mobility is due to the high *V*_*G*_-induced carrier density^31^ of ~10^13^ cm^−2^ to screen out charged defects. This is also supported by the higher mobility with larger *V*_*G*_^7^, where induced carrier density increases with *V*_*G*_. In the thicker 7 ~ 20 nm SnO_2_ devices with poor pinch off, the mobility is lowered by extra parallel conduction from non-depleted bulk SnO_2_. The mobility is lowered in 3.5 nm SnO_2_ TFT due to stronger roughness scattering from top surface. Here the SnO_2_ surface roughness is 0.39 nm, close to the HfO_2_ roughness of 0.41 nm.

**Figure 2:**
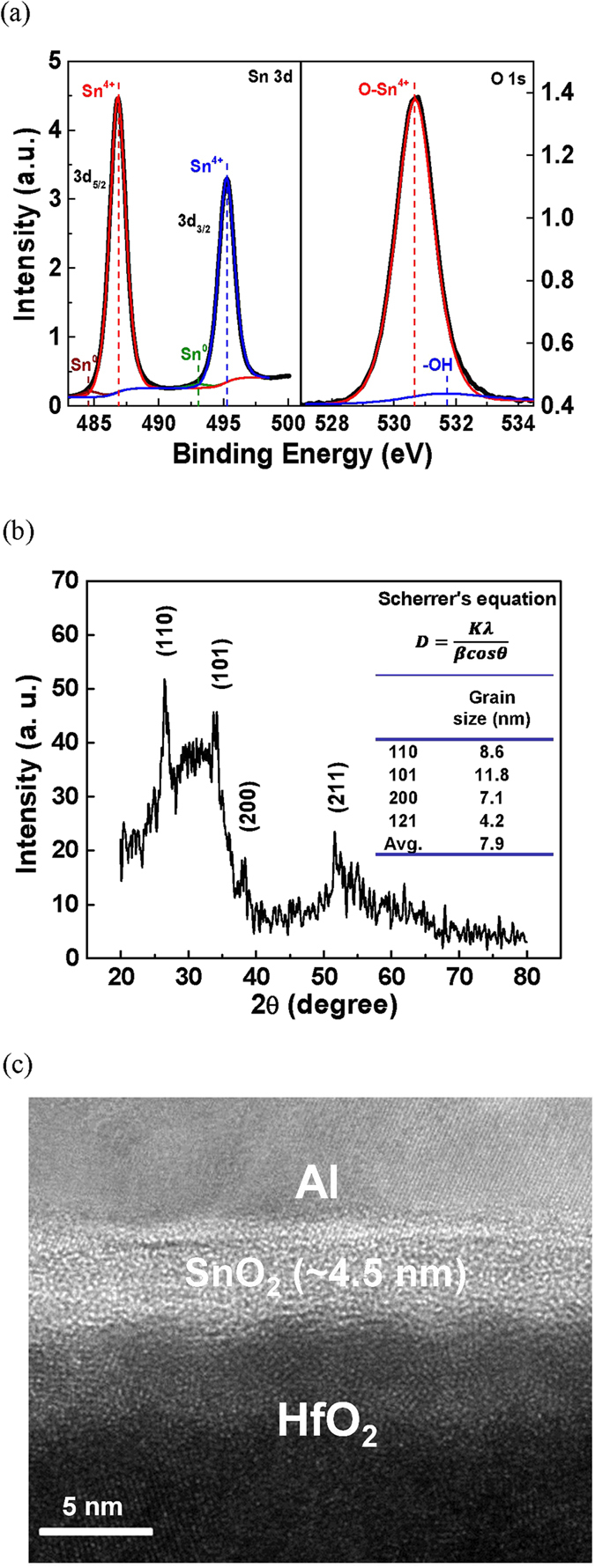
(**a**) XPS, (**b**) XRD, and (**c**) TEM analysis of SnO_2_ formed on HfO_2_/n^+^-Si. The SnO_2_ thickness is 20 nm for XPS and XRD analysis, while 4.5 nm for TEM.

The high mobility SnO_2_ TFT was further investigated using material analysis. Figure 2(a) shows the X-ray photoelectron spectroscopy (XPS) spectra from the Sn 3d and O 1s core level of the SnO_2_ thin film. The Sn 3d peak corresponded to the oxidation state of Sn^4+^, and the O 1s peak was attributed to the O-Sn and O-H bonds. Thus, the chemical composition was determined to be Sn^4+^O_2_^2−^. The X-ray diffraction spectroscopy (XRD) pattern in Fig. 2(b) reveals the presence of a rutile phase in SnO_2_. An average grain size of 7.9 nm was obtained using the Scherrer’s equation. Figure 2(c) shows the cross-sectional transmission electron microscopy (TEM) image of the SnO_2_/HfO_2_ stack. A relatively uniform SnO_2_ layer with an ultra-thin thickness of 4.5 nm was observed.

**Figure 3:**
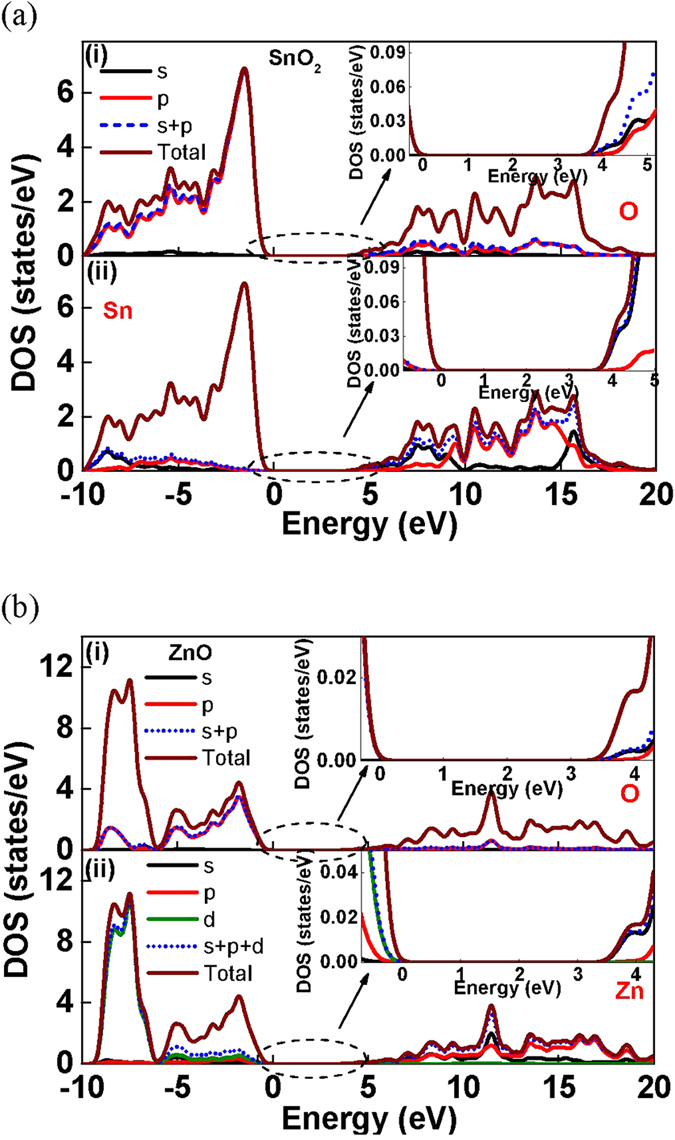
(**a**) DOS of (i) O, and (ii) Sn in SnO_2_, and (**b**) DOS of (i) O, and (ii) Zn in ZnO.

To thoroughly understand the cause of the high mobility in SnO_2_ TFT, first principle quantum-mechanical calculations were used to investigate the electronic structures of SnO_2_ and ZnO; ZnO has been extensively studied using the localized density functional theory (DFT) to reveal the mechanism that leads to its high mobility. The structures of both SnO_2_ and ZnO semiconductors were successfully obtained using local density approximation plus *U* (LDA+*U*) method with appropriate *U*^*p*^ and *U*^*d*^ value. The LDA+*U* method compensates for the underestimation of the bandgap caused by a strong self-interaction by the DFT. The bandgaps of SnO_2_ and ZnO were calculated to be 3.68 and 3.39 eV (Figure S1(a) and S1(b)), respectively, which are consistent with the experimental values of 3.6 and 3.4 eV, respectively. The contribution of each orbital in the conduction band minimum (CBM) of SnO_2_ was investigated using density of state (DOS) analysis. The energy of valence band minimum was set to zero for convenience. As shown in Fig. 3(a), the upmost valence band was predominated by the O 2p orbitals, and the contribution from Sn was mostly from 5p orbitals. The lower conduction states near CBM were mostly derived from Sn 5s orbitals, whereas the O 2p orbitals contributed only in higher energy states. The Sn 4d orbitals did not give rise to the electron conducting property of SnO_2_ because the antibonding interaction between Sn 4d and O 2p orbitals led only to the slight mixing of states at a deep valence band level. The DOS results of ZnO (Fig. 3(b)) were similar to those of SnO_2_. The major difference between the valence bands of SnO_2_ and ZnO is the contribution of d orbitals. The upper valence bands (from −6 to 0 eV) were composed of primarily of O 2p orbitals and slight mixing states from Zn 4s, 4p and 3d, whereas Zn 3d orbitals dominated in deeper states. In conduction band, Zn 4s was the major component near CBM while O 2p orbitals had little contribution at levels lower than 5 eV. Therefore, the high mobility of SnO_2_ was attributed to the overlapping of s-orbitals, as with ZnO, although it had ns^2^np^2^ configuration. This is further supported by the charge density distribution of SnO_2_ shown in Figure S2(a), which has highly overlapped orbitals similar with those of ZnO in Figure S2(b). The highly overlapped orbitals of SnO_2_ is related to large atomic radius, one row below Zn in the periodic table. From the results of DOS and charge density distribution, the higher mobility than the state-of-the-art ZnO TFTs^32^,^33^ is attributed to the highly overlapped s-orbitals of SnO_2_.

The carrier effective mass is a major factor that may explain the higher mobility of SnO_2_ than that of ZnO. The high electron mobility of n-type materials is caused by a deep curvature in CBM of band structure shown in Figure S1(a) and S1(b), which leads to a low effective mass of electrons. The calculated electron effective mass of SnO_2_ was approximately 20% lighter than that of ZnO, indicating a faster electron transport in the SnO_2_ conduction band.

In conclusion, this SnO_2_ TFT device had a considerably high mobility, high *I*_*ON*_*/I*_*OFF*_, low *SS*, low operation voltage, and ultra-thin thickness. The low operation voltage is due to the high-κ gate dielectric with a high capacitance density. The low SS indicates the good gate dielectric and SnO_2_ interface. The high *I*_*ON*_/*I*_*OFF*_ is related to the high mobility to increase *ION* and the ultra-thin thickness to decrease *I*_*OFF*_, where the high mobility is caused by strongly overlapped s-orbitals.

## Methods

The SnO_2_ TFTs were fabricated on a heavily doped n-type silicon (100) substrate. The 40-nm-thick high-κ gate HfO_2_ and 20 ~ 3.5-nm-thick SnO_2_ films were deposited by physical vapor deposition. Thicker 20 nm SnO_2_ film was also deposited for X-ray photoelectron spectroscopy (XPS) and X-ray diffraction (XRD) pattern analysis. Then the high-κ layer and SnO_2_ film was annealed at 400^o^C. Finally, the Al source-drain electrodes were thermally evaporated and patterned. The gate length and width are 50 ~ 150 μm and 500 μm, respectively. Therefore, the maximum process temperature for this device is 400^o^C. The fabricated devices were characterized by XPS, XRD, TEM, *C-V*, and *I-V* measurements. All quantum-mechanical calculations were performed by Cambridge Sequential Total Energy Package (CASTEP) code. Structural optimization was performed on each model prior to calculating their electrical properties. The LDA+*U* method is known to correct the strong correlation of metal oxides and is proven to be quite effective for ZnO. The calculations were carried out by using generalized gradient approximation (GGA) with LDA+*U*.

## Additional Information

**How to cite this article**: Wei Shih, C. *et al.* Remarkably high mobility ultra-thin-film metal-oxide transistor with strongly overlapped orbitals. *Sci. Rep.* **6**, 19023; doi: 10.1038/srep19023 (2016).
